# Comparative Analysis of Intestinal Microbiota in Wild, Domesticated, and Cultured *Gymnocypris potanini firmispinatus*

**DOI:** 10.3390/biology13120983

**Published:** 2024-11-28

**Authors:** Baoshan Ma, Jiaqi Zhang, Dapeng Li, Zhipeng Chu, Jieya Liu, Jiali Jin, Liqiao Zhong

**Affiliations:** 1 National Agricultural Science Observing and Experimental Station of Chongqing, Yangtze River Fisheries Research Institute, Chinese Academy of Fishery Science, Wuhan 430223, China; baoshanma@yfi.ac.cn (B.M.); 13971510701@163.com (J.Z.); chuzhipeng@yfi.ac.cn (Z.C.); 2 College of Fisheries, Huazhong Agricultural University, Wuhan 430070, China; ldp@mail.hzau.edu.cn (D.L.); dkyliujieya@163.com (J.L.); 3 College of Life Sciences and Technology, Tarim University, Alar 843300, China

**Keywords:** intestinal microbiota, *Gymnocypris potanini firmispinatus*, wild group, domesticated group, cultured group

## Abstract

The impact of the living environments on the intestinal microbiota of *Gymnocypris potanini firmispinatus* was studied by analyzing intestinal microbiota diversity, composition, and potential function among wild, domesticated, and cultured groups. The results showed that the fish living in the wild environment exhibited the highest alpha diversity of intestinal microbiota. Intestinal microbial communities in the three groups clustered by living environment, with domesticated and cultured groups showing higher similarity. The dominant phylum in the wild group was Proteobacteria, whereas Fusobacteria and Proteobacteria were the predominate bacteria in the domesticated and cultured groups. RsaHF231, Actinobacteria, Fusobacteria, Cyanobacteria, and Firmicutes were the five key bacteria differentially expressed among the groups. Functional prediction revealed significant variation in the L-cysteine degradation III pathway (PWY-5329) between wild and domesticated groups, and in the vitamin E biosynthesis pathway (PWY-1422) between wild and cultured groups (*p* < 0.05). These findings highlight the differences in microbiota communities between wild and farmed fish, which are essential for enhancing the cultivation of *G. p. firmispinatus* and for the formulation of specific probiotics and functional feeds.

## Simple Summary

Significant differences in the intestinal microbiome composition of wild, domesticated, and cultured *Gymnocypris potanini firmispinatus* were identified at the phylum and genus levels, reflecting the contrast between wild and farming conditions. Greater richness and diversity in the intestinal microbiota were observed in the wild group, while domesticated and cultured groups exhibited lower richness and diversity. The clustering of intestinal microbial communities was distinctly associated with their respective environments, with a higher degree of similarity noted between domesticated and cultured groups. Our findings further highlighted the influence of different environments and food resources in shaping the functional profiles of fish intestinal microbiota.

## 1. Introduction

Intestinal microbiota is regarded as essential components of an organism [1]. It is characterized by its complexity, diversity, and abundance in fish, forming a superorganism that interacts with its host [2]. Normally, the intestinal microbiota and the host jointly maintain the typical physiological functions of fish. Beneficial bacteria, during metabolic processes, secrete digestive enzymes and other substances that enhance the digestion and absorption in fish or serve directly as sources of nutrients [3,4,5,6]. Moreover, intestinal microbiota is capable of activating the immune response in fish and controlling the proliferation of pathogens in the intestine [7,8,9]. The equilibrium of intestinal microbiota is closely linked to the health of the host [10]. Conversely, a disturbance in this equilibrium, known as intestine dysbiosis, results in an excessive increase in pathogenic bacteria and is associated with health issues in individuals with both communicable and noncommunicable diseases [11,12]. Intestine dysbiosis is defined as an imbalance in the taxonomic composition and metagenomic function of the microbial community. This condition may arise from an overgrowth of potentially harmful components of the intestinal microbiota, termed the “bloom of pathobionts”, or from a reduction or complete disappearance of normally residing members, referred to as the “loss of commensals” [12].

The intestinal microbiota of fish is influenced by diet, sex, physiological status, environment, genetics, and other factors [5,13,14,15]. It has been shown that the structure of microbiota communities in fish intestines can be altered by various living environments [2]. Strong differences were found in the intestinal microbiota composition at the phylum level, with Proteobacteria and Firmicutes being the most abundant in wild and aquaculture conditions of *Paralichthys adspersus*, respectively [16]. In wild conditions, lipid metabolism associated with unsaturated fatty acid synthesis is notably present, whereas in aquaculture conditions, the metabolism of terpenoids and polyketides is significant in *Genypterus chilensis* [2]. Therefore, by comparing the composition, diversity, and function of the intestinal microbiota in wild and cultured fish, it can be concluded that environmental conditions are important factors affecting the structure of fish intestinal microbiota [2,14,17].

Domesticating new fish poses challenges in controlling biological and abiotic factors to imitate the life cycles and behaviors observed in wild fish [18]. Successful domestication depends on species biology, feeding techniques, nutritional needs, and captive breeding control. Central to domestication is altering environmental conditions and feeding habits, necessitating fish adaptation, including adjusting intestinal microbiota to artificial diets. Nutrient intake is vital for survival, with intestine microbes playing a crucial role in this process [2]. Intestinal microbiota has been identified as a significant factor influenced by captivity in fish domestication, and its modulation may be crucial in the future for controlling fish diseases in aquaculture [2,16].

*Gymnocypris potanini firmispinatus*, an endemic schizothoracine species, is found exclusively in the Jinsha River and its tributaries. This species inhabits cold-water environments in plateau rivers and primarily consumes aquatic insects [19]. Compared with other schizothoracine fishes, the muscle of *G. p. firmispinatus* is noted for its superior freshness and tenderness, making it highly valuable economically. It is also rich in functional amino acids and fatty acids, including lysine, eicosapentaenoic acid (EPA), and docosahexaenoic acid (DHA) [20]. However, factors such as overfishing and hydropower development have significantly contributed to the depletion of natural resources of *G. p. firmispinatus*, which has been classified as a key protected species in Sichuan Province, China. In recent years, efforts have successfully domesticated, bred, and cultured *G. p. firmispinatus* to protect and expand its population. However, research on *G. p. firmispinatus* remains scarce, focusing mainly on its ecological and biological characteristics [19,20,21,22]. Therefore, to comprehensively understand microbiota differentiation among *G. p. firmispinatus* populations under different living conditions, 16S rDNA sequencing was employed to analyze the diversity, composition, and potential functions of intestinal microbiota in wild, domesticated, and cultured groups. This study aims to enhance the successful culture of this endemic and recently developed fish species.

## 2. Materials and Methods

### 2.1. Sample Collection

The wild, domesticated, and cultured groups of *G. p. firmispinatus* were designed in this study. Wild individuals were captured using trap nets (mesh of 0.5 cm, 5 m long or mesh of 1 cm, 10 m long) in December 2021 from tributaries of the Anning River at an altitude of 1954–2077 m. The flow velocity ranged from 1.1 to 1.2 m/s, with water depths of 0.2–0.5 m and channel widths of 5–7 m. Domesticated and cultured *G. p. firmispinatus* were obtained in January 2022 from the Liyuan breeding station, Yunnan province, China. The domesticated individuals came from wild fish caught in tributaries of the Jinsha River in December 2019 and were subsequently domesticated at the Liyuan breeding station. The cultured group was the offspring artificially bred at the Liyuan breeding station in March 2018, which was close to or had reached sexual maturity. Both the domesticated and cultured groups were reared in a recirculating aquaculture tank of 2 m in diameter and 1 m in height. The aquaculture water was obtained from the Jinsha River, and the water temperature was stably maintained at 9–11 °C. The domesticated group was initially provided with artificial compound feed (4.0) once daily in the morning. From December 2021, this group received artificial compound feed (4.0) twice daily, in the morning and at nightfall, supplemented with mealworms in the morning to enhance breeding as parent fish. The cultured group was fed artificial compound feed (2.0) once daily in the morning. The composition of the feed is presented in Table 1.

After capture, the fish were anesthetized. The hind intestine contents were collected aseptically, placed into 2 mL frozen tubes, rapidly frozen in liquid nitrogen, and immediately transferred to a −80 °C freezer for storage. The 4–9 vertebrae of each specimen were taken for subsequent age identification. The age was determined by observing the annuli on the vertebrae [23]. Based on the vertebra annuli of specimens, 4-year-old *G. p. firmispinatus* were selected for analysis, comprising 10 wild specimens, 5 domesticated specimens, and 8 cultured specimens.

### 2.2. DNA Extraction and Sequencing

The intestine contents from the three groups were taken for total genomic DNA extraction. Total genomic DNA samples were extracted using the OMEGA Soil DNA Kit (M5635-02) (Omega Bio-Tek, Norcross, GA, USA). The extracted DNA was assessed using 0.8% agarose gel electrophoresis and quantified with a Nanodrop NC2000 spectrophotometer (Thermo Fisher Scientific, Waltham, MA, USA). PCR amplification targeting the V4−V5 region of the bacterial 16S rRNA gene was conducted using a specific primer with a barcode, with a total of 25 cycles performed.

The bacterial universal primers 515F (5′-GTGCCAGCMGCCGCGGTAA-3′) and 907R (5′-CCGTCAATTCCTTTGAGTTT-3′) were used to amplify the V4−V5 variable region of bacterial 16S rRNA. Sample-specific 7-bp barcodes were incorporated into the primers for multiplex sequencing. The total volume of the PCR system was 25 μL, with 0.25 μL of Q5 high-fidelity DNA polymerase, 5 μL of 5*Reaction Buffer, 5 μL of 5*High GC Buffer, 2 μL of dNTP (10 mM), 2 μL of template DNA, 1 µL of each primer (10 µM), and 8.75 μL ddH_2_O. The PCR amplification process involved initial denaturation at 98 °C for 5 min, followed by 25 cycles of 98 °C for 30 s, 55 °C for 30 s, 72 °C for 45 s, and a final extension at 72 °C for 5 min. The PCR products of the specimens were mixed, and 2% agarose gel electrophoresis was subsequently performed. The target electrophoretic bands were excised and purified using a Qiagen Gel Extraction Kit (Qiagen, Hilden, Germany), and quantified with the Quant-iT PicoGreen dsDNA Assay Kit (Invitrogen, Carlsbad, CA, USA). After the individual quantification step, amplicons were pooled in equal amounts, and pair-end 2 × 250 bp sequencing was performed using the Illlumina NovaSeq platform with NovaSeq 6000 SP Reagent Kit (500 cycles) at Shanghai Personal Biotechnology Co., Ltd. (Shanghai, China).

### 2.3. Sequence Analysis

Microbiome bioinformatics were performed with QIIME2 2019.4 [24] with slight modification according to the official tutorials (https://docs.qiime2.org/2019.4/tutorials/ accessed on 19 October 2024). Briefly, raw sequence data were demultiplexed using the demux plugin following by primers cutting with cutadapt plugin [25]. Sequences were then quality filtered, denoised, merged and chimera removed using the DADA2 plugin [26]. The length distribution of the high-quality sequences was analyzed using the classify-sklearn algorithm. Each amplicon sequence variant (ASV) or representative sequence of the operational taxonomic unit (OTU) was taxonomically annotated using a pretrained naive Bayes classifier with default parameters. Finally, the “qiime feature-table rarefy” function was applied, setting the extraction depth to 95% of the minimum sample sequence size. The above analysis was conducted using QIIME2 (version 2019.4) software [27].

### 2.4. Diversity Indices and Statistical Analysis

Intestinal microbial richness was evaluated using the Chao1 and observed species indices, while diversity was assessed with the Shannon and Simpson indices [2]. Evolutionary diversity was calculated using Faith’s PD index [28]. Nonparametric Kruskal-Wallis test, was applied to detect significant differences in the alpha diversity indices among the three groups. The threshold for statistical significance was set at 0.05. The above alpha diversity indices were analyzed by using QIIME2and the R software (version 3.6.1).

Beta diversity analysis utilized nonmetric multidimensional scaling (NMDS) based on the Bray–Curtis distance matrix to assess differences in microbial community composition among the three groups. Cluster analysis was performed using the unweighted pair-group method with arithmetic means (UPGMA) algorithm in the R software, and visualization was conducted using the “ggtree” package. Subsequently, based on the Bray–Curtis distance, comparisons among the three groups were performed via PERMANOVA and ANOSIM.

The species composition was analyzed by heatmap in the R software. Differential marker species among the wild, domesticated, and cultured groups were identified through random forest analysis and nested stratified cross-validation using QIIME2 software. Nonparametric Kruskal–Wallis tests, Wilcoxon rank sum tests, and linear discriminant analysis (LDA) were subsequently performed to identify taxa with significant differences in relative abundance among the three groups via LEfSe package in Python software (version 2.7). The threshold of the logarithmic LDA score was set as 4.0 for distinguishing significant features.

### 2.5. Prediction of Molecular Functions Using PICRUSt2

The functional pathways of the intestinal microbiota were analyzed using the metabolic pathway database MetaCyc (https://metacyc.org/ accessed on 19 October 2024). Abundance values for metabolic pathways were normalized, and the average abundances of second-level pathways were calculated. In the R software, the “metagenomeSeq” package was utilized to fit the distribution of each metabolic pathway using the “zero-inated log-normal model”. These fitted models were used to determine the significant differences of the third-level pathways among the three groups, with a significance level of *p* < 0.05 [29]. PICRUSt2 (version 2.2.0-b; https://github.com/picrust/picrust2/wiki accessed on 19 October 2024) and the R software were employed for this analysis.

## 3. Results

### 3.1. Sequencing Depth

**Figure 1:**
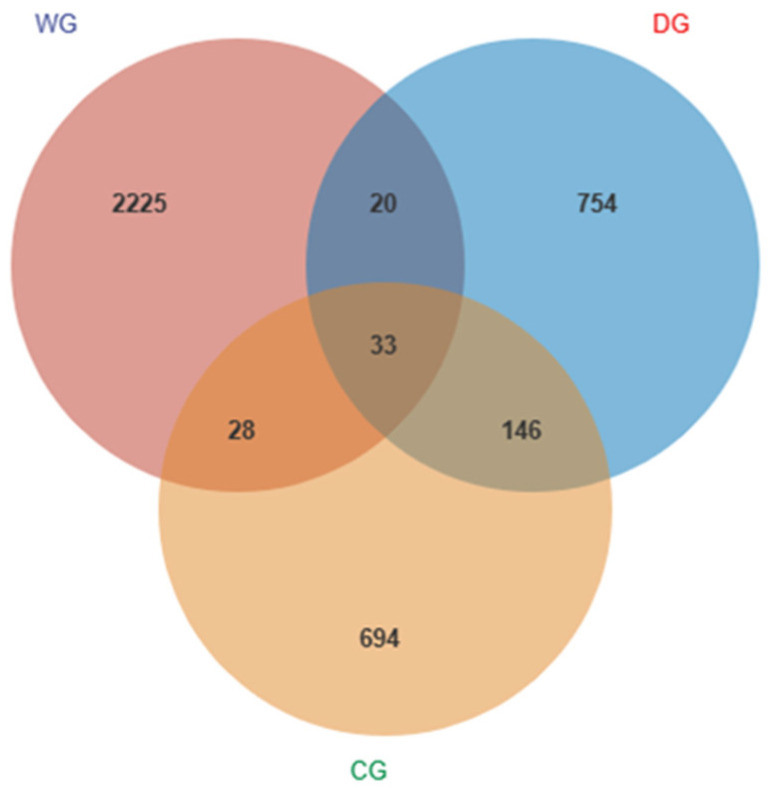
ASV Venn analysis chart. WG: wild group; CG: cultured group; DG: domesticated group.

A total of 1,461,882 valid sequences were obtained. The number of the sequences ranged from 40,528 to 75,761 per sample, with an average number of 63,560. According to the Wayne chart analysis, the samples contained 2306 ASVs in the wild group, while 953 and 901 ASVs in the domesticated and cultured groups, respectively. Among which, 33 ASVs were shared in the three groups. The wild fish shared 61 and 53 ASVs with the cultured and domesticated groups, respectively, whereas the cultured and domesticated groups shared 179 ASVs (Figure 1).

### 3.2. Diversity of Intestinal Microbial Communities

**Figure 2:**
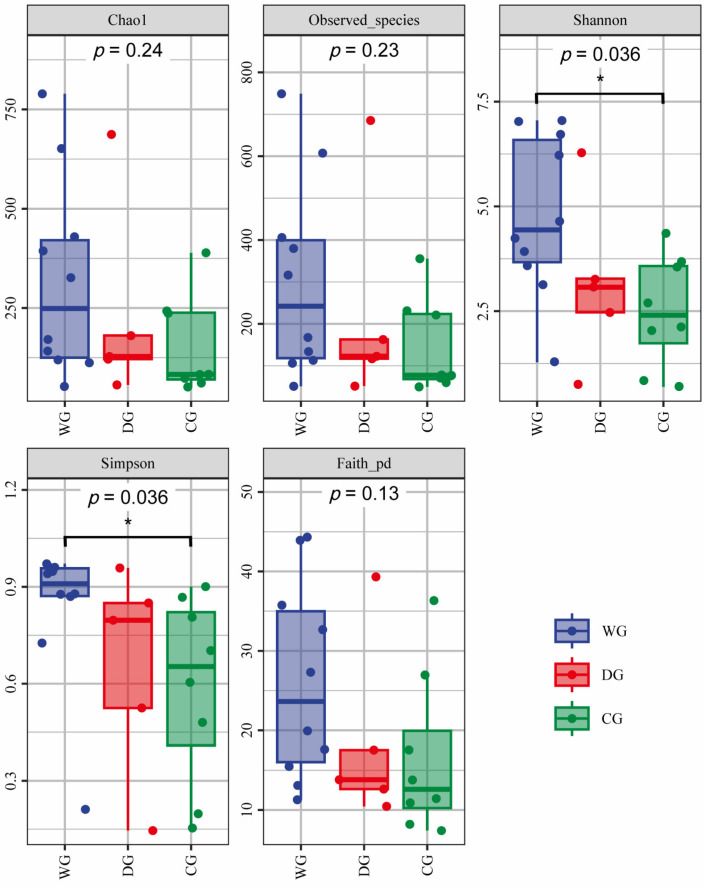
Box plots of alpha diversity of intestinal microbiota in wild, domesticated, and cultured *G. p. firmispinatus*. Dunn’s test: * means *p* < 0.05. WG: wild group; CG: cultured group; DG: domesticated group.

Among the three groups, the intestinal microbiota in the wild group exhibited the highest values for all five alpha diversity indices (Chao1, observed species, Shannon, Simpson, and Faith-PD indices) (Figure 2). Although the diversity indices in the wild group were higher than those in the domesticated group, the differences were not statistically significant (*p* > 0.05). The indices of Chao1, observed species, and Faith-PD showed no significant differences between the wild and cultured groups, whereas the Shannon and Simpson indices did exhibit significant differences (*p* < 0.05). There were no significant differences in the five alpha diversity indices between the domesticated and cultured groups (*p* > 0.05). The wild group demonstrated higher richness and diversity in intestinal microbiota, reflecting greater ecological resilience, whereas the domesticated and cultured groups exhibited reduced diversity and increased similarity among individuals.

**Figure 3:**
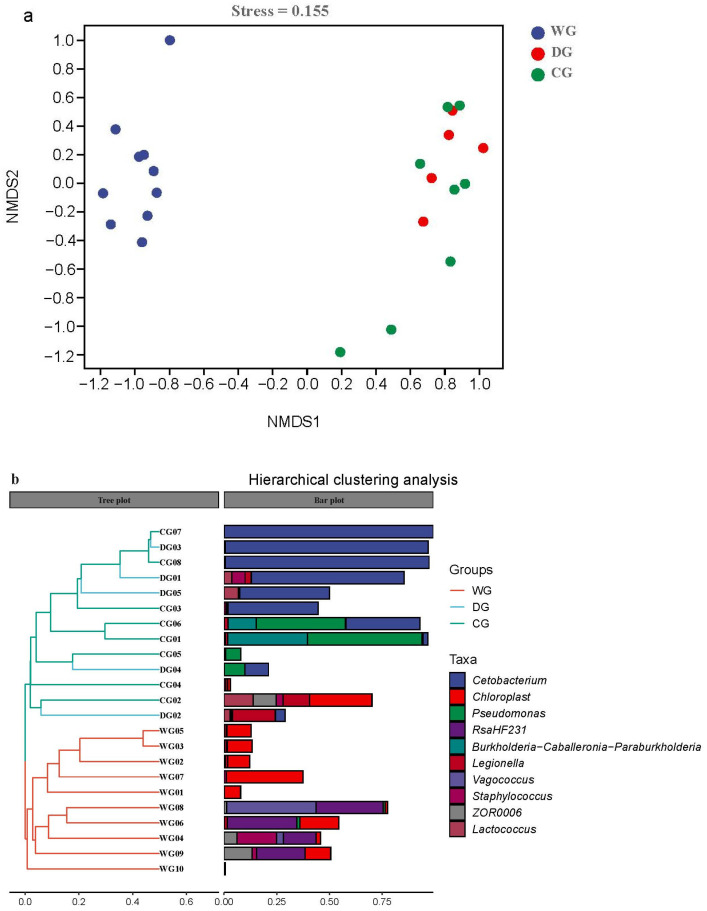
Visualized samples by NMDS based on Bray–Curtis distances (**a**) and hierarchical clustering analysis (**b**) in the intestinal microbiota of wild, domesticated, and cultured *G. p. firmispinatus*. WG: wild group; CG: cultured group; DG: domesticated group.

The NMDS analysis of beta diversity indicated that the intestinal microbiota communities of the domesticated and cultured groups were similar (Figure 3a). PERMANOVA and ANOSIM results confirmed significant differences in intestinal microbiota community structure between the wild group and the other two groups (*p* < 0.01), while no significant difference was found between the domesticated and cultured groups (*p* > 0.05, Table 2). The dendrogram demonstrated that the wild group was different from the other two groups and formed a separate cluster, while the domesticated and cultured groups came under another cluster. Notably, within the cultured group, obvious variation in the intestinal microbiota communities was observed among individuals (Figure 3b).

### 3.3. Differences in the Microbiota Composition Among the Three Groups

**Figure 4:**
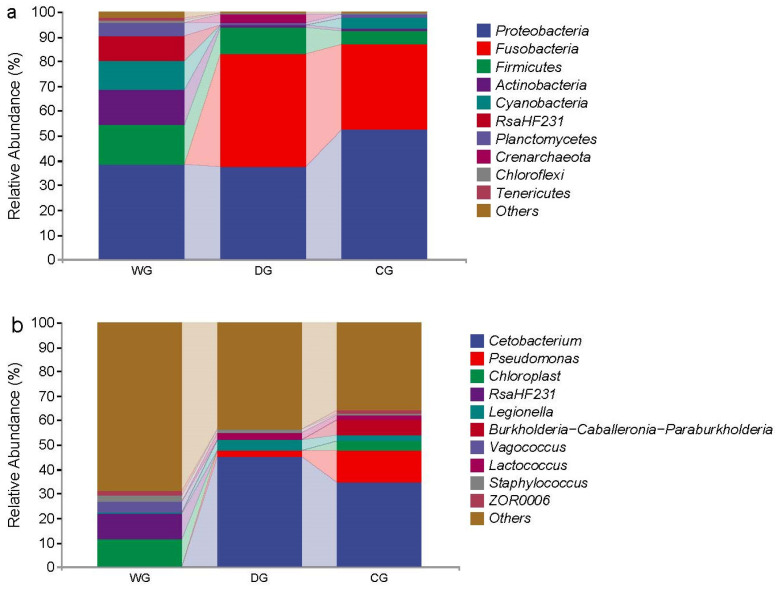
Composition of intestinal microbiota abundance at the phylum level (**a**) and genus level (**b**). WG: wild group; CG: cultured group; DG: domesticated group.

At the phylum level, the intestinal microbiota of the wild group predominantly consisted of Proteobacteria (38.26%), Firmicutes (16.25%), Actinobacteria (11.87%), and Cyanobacteria (11.87%). In contrast, the dominant phyla in the domesticated group were Fusobacteria (45.38%), Proteobacteria (37.48%), and Firmicutes (10.89%). The cultured group showed Proteobacteria (52.23%) and Fusobacteria (34.50%) as the major phyla (Figure 4a).

At the genus level, *Chloroplast* (11.16%) and *RsaHF231* (10.42%) were the predominant bacteria in the wild group, while *Cetobacterium* (45.38%) was dominant in the domesticated group, followed by *Legionella* (4.71%) and *Pseudomonas* (2.02%). In the cultured group, *Cetobacterium* (34.50%) and *Pseudomonas* (13.04%) were the dominant genera, followed by *Burkholderia*-*Caballeronia*-*Paraburkholderia* (6.49%) (Figure 4b).

**Figure 5:**
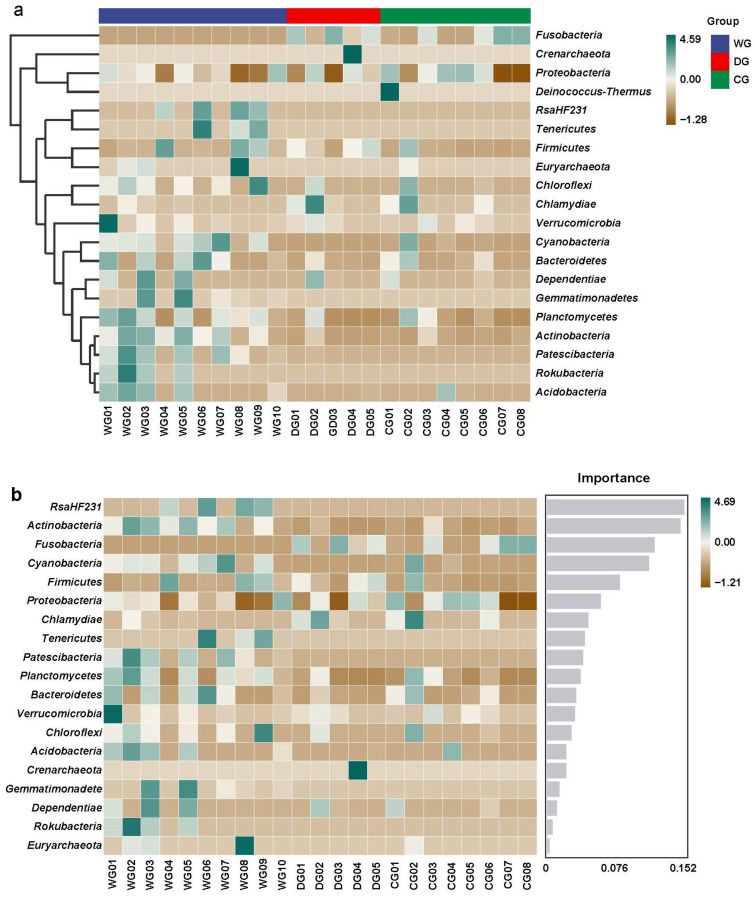
A heat map with the abundance of the top 20 OTUs (**a**) and random forest analysis (**b**) of the intestinal microbiota among wild, domesticated, and cultured *G. p. firmispinatus*. WG: wild group; CG: cultured group; DG: domesticated group.

A heatmap was generated using the average abundance data of the top 20 phyla, illustrating significant differences in species composition among the three groups (Figure 5a). In the wild group, the most abundant phyla included RsaHF231, Tenericutes, Firmicutes, Cyanobacteria, Bacteroidetes, Gemmatimonadetes, and Actinobacteria. Conversely, the domesticated and cultured groups predominantly exhibited Fusobacteria and Proteobacteria in their intestinal microbiota compositions.

The results of the random forest map showed that among the top 20 phyla with high relative abundances, 5 phyla were differentially expressed among the three groups, with scores higher than 0.076, namely, RsaHF231, Actinobacteria, Fusobacteria, Cyanobacteria, and Firmicutes (Figure 5b).

**Figure 6:**
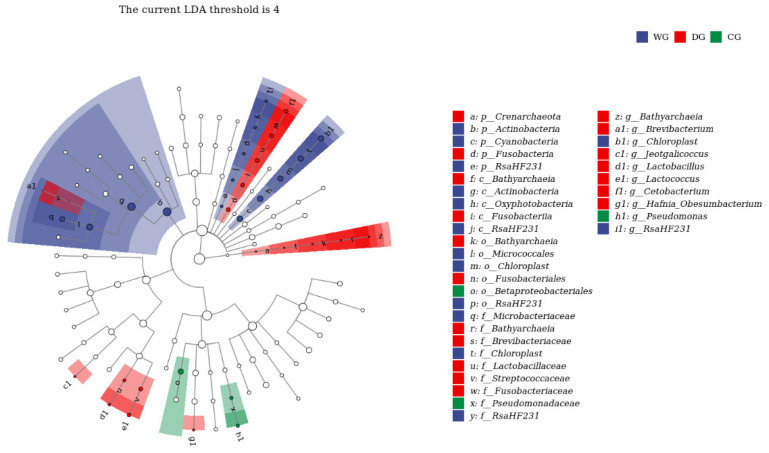
LefSe analysis of the intestinal microbiota among wild, domesticated, and cultured *G. p. firmispinatus*. WG: wild group; CG: cultured group; DG: domesticated group.

LefSe analysis identified 35 distinct taxa of intestinal microbiota among the wild, domesticated, and cultured groups, encompassing five phyla, five classes, six orders, nine families, and ten genera. In the wild group, phyla with high relative abundances included Actinobacteria, Cyanobacteria, and RsaHF231, with dominant genera such as *Chloroplast* and *RsaHF231*. In the domesticated group, the phyla Crenarchaeota and Fusobacteria were prominent, along with genera including *Bathyarchaeia*, *Brevibacterium*, *Jeotgalicoccus*, *Lactobacillus*, *Lactococcus*, *Cetobacterium*, and *Hafnia-Obesumbacterium*. In the cultured group, the genus *Pseudomonadaceae* exhibited a high relative abundance (Figure 6).

### 3.4. Functional Pathways with Significant Differences Among the Three Groups

**Figure 7:**
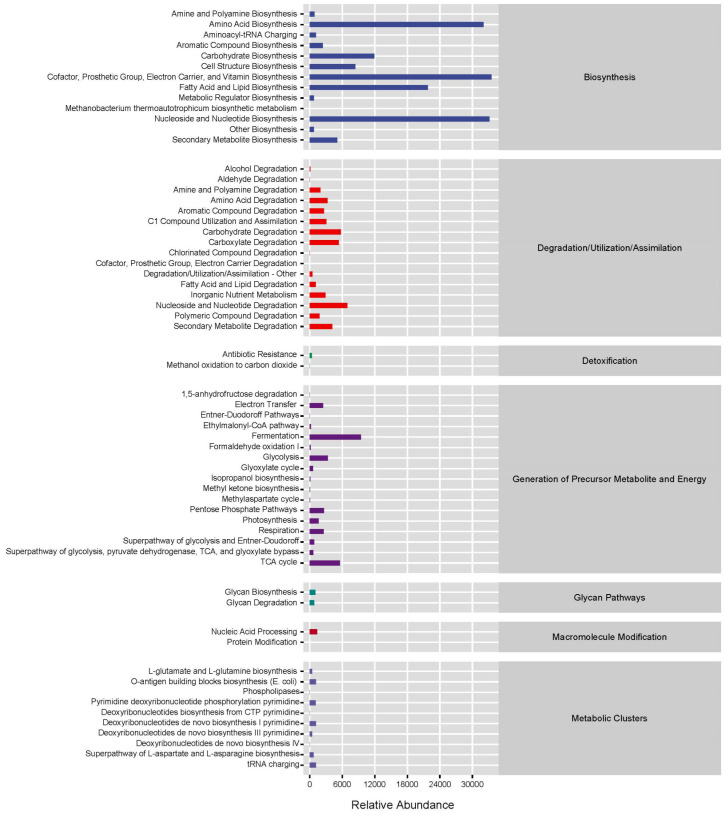
Statistics of metabolic pathways of *G. p. firmispinatus* intestinal microbiota.

The analysis revealed that in the first metabolic pathways of the MetaCys database, the intestinal microbiota of all three groups primarily engaged in biosynthesis, with a smaller portion involved in degradation, utilization, assimilation, and the generation of precursor metabolites and energy. Among the biosynthetic pathways in the second-level metabolic pathways, the highest relative abundances were observed in amino acid biosynthesis, biosynthesis of cofactors, prosthetic groups, electron carriers, and vitamins, fatty acid, and lipid biosynthesis, as well as nucleoside and nucleotide biosynthesis (Figure 7).

**Figure 8:**
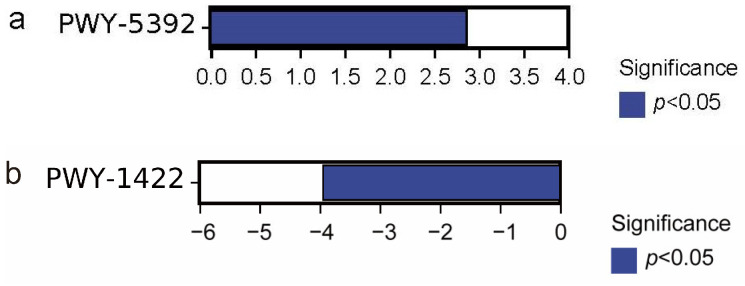
Analysis of differences in metabolic pathways of intestinal microbiota among the three groups. (**a**) WG vs. DG; (**b**) WG vs. CG. WG: wild group; CG: cultured group; DG: domesticated group.

Significant difference in third-level metabolic pathways was observed in the L-cysteine degradation III pathway (PWY-5392) between the wild and domesticated groups (*p* < 0.05, Figure 8a). Additionally, a significant difference was found in the vitamin E biosynthesis pathway (PWY-1422) between the wild and cultured groups (*p* < 0.05, Figure 8b). No significant difference was observed between the domesticated and cultured groups (*p* > 0.05).

## 4. Discussion

### 4.1. Diversity of the Intestinal Microbiota

Among the three groups, th intestinal microbiota of the wild group exhibited the highest values for all five alpha diversity indices. The elevated microbial diversity in the gut of wild fish is likely attributable to the complexity and variability of natural water environments and food conditions [30]. Nevertheless, the analysis of alpha diversity revealed no significant differences in the richness and diversity of the intestinal microbiota between the wild and domesticated groups, which aligns with findings in wild and domesticated *Schizothorax o’connori* [31] and previous studies on *P. adspersus* [16] and *G. chilensis* [2]. These results suggest that transitioning from wild to cultured environments may have limited impact on intestinal microbiota diversity and richness. However, there may be alterations in the species composition and abundance of intestinal microbiota to some extent. In this study, the domesticated group comprised 4-year-old fish captured in December 2019, after spending two years in a wild environment. Their intestinal microbiota may have retained similarities with the wild group, which accounts for the absence of significant differences in diversity. Nevertheless, changes in aquaculture conditions and diet could gradually influence microbiota composition and abundance.

Restivo et al. [32] reported that transferring wild fish into artificial ponds altered the alpha and beta diversity of the microbiota. The beta diversity of the intestinal microbiota in *Salmo salar* was altered when the fish were fed different diets [33]. This study demonstrated that the intestinal microbiota of *G. p. firmispinatus* was influenced by diet in recirculating aquaculture systems. Schmidt et al. [34] similarly found that the microbiota of *S. salar* was affected by diet in such systems. These findings are similar to those of other recent studies comparing wild and farmed fish, such as *P. adspersus* [16], *Seriola lalandi* [17], *S. salar* [35], and *Sparus aurata* [36].

The intestinal microbiota of wild *G. p. firmispinatus* showed a distinct clustering pattern while the intestinal microbiota of domesticated and cultured groups reared in recirculating aquaculture systems clustered together. This clustering indicates different microbiota compositions between wild and farmed environments. Yang [37] also observed the distinct clustering of intestinal microbiota in *Schizopygopsis malacanthus* from different river systems based on their respective habitats.

Habitat conditions, dietary composition, and other factors profoundly influence the establishment of microbial communities in animal intestines [14]. According to Zhang [38], the environments where fish live impose selective pressures, prompting gradual adaptation. Dhanasiri et al. [39] observed a reduction in the diversity of fish intestinal microbiota over time with increased reliance on artificial feeding. In this study, while the intestinal microbiota structures of the domesticated and cultured groups were similar, they differed significantly from those of the wild group. This suggests that the diversity of intestinal microbiota in domesticated *G. p. firmispinatus* gradually declined during the feeding process before stabilizing.

### 4.2. Species Composition of the Intestinal Microbiota

Our findings suggest that wild *G. p. firmispinatus* possess a distinct microbiota composition compared to domesticated and cultured fish. This finding highlighted the impact of food resources and varying environmental conditions on the composition of fish intestinal microbiota. In the present study, wild fish primarily consumed aquatic insects [19], while domesticated fish were fed artificial compound feed supplemented with mealworms, and cultured fish relied solely on artificial compound feed (Table 1). Moreover, the water quality in natural environments is likely more conducive to fish health compared to conditions in recirculating aquaculture systems. This difference suggests that conditions in aquaculture systems lead to the establishment of different bacterial communities in the intestine compared to wild specimens, likely influenced by controllable factors such as population density, diet, and water quality, which can vary considerably depending on the fish’s environment [2].

The intestinal microbiota of the wild group exhibited higher diversity, primarily dominated by Proteobacteria. In contrast, the microbiota in the intestines of the domesticated and cultured groups were comparatively simpler, with Proteobacteria and Fusobacteria as the dominant taxa. Proteobacteria, followed by Fusobacteria and Firmicutes, are commonly dominant phyla in the intestinal microbiota of various freshwater fish species such as *Etheostoma caeruleum* [32], *Carassius auratus gibelio* [40], *Carassius auratus* [41], and *Siganus oramin* [6]. Many pathogenic bacteria within the Proteobacteria phylum are conditional, and their proportion in the intestinal tract can reflect the stability of the microecology and the structure of the intestinal microbial community, serving as a microbial marker of intestinal microbial imbalance [42]. The balance between microbes and the host intestine in fish is maintained through selective interactions with various microbiota [6,43], which may lead to Proteobacteria becoming the dominant phylum in the intestinal microbiota of *G. p. firmispinatus*. Firmicutes are of significant interest in aquaculture due to their inclusion of genera, such as lactic acid bacteria, which are associated with pathogen protection and the enhancement of immune system development, thereby improving host resistance to diseases [2]. Additionally, for farmed fish, the phylum Fusobacteria is considered a core component of their microbiota, consistent with findings by Mugetti et al. [30]. The prominent presence of Fusobacteria is likely linked to animal-protein-based diets, as highlighted by studies on reared teleosts fed fishmeal-based diets [30]. In the present study, the Fusobacteria identified were predominantly attributed to the genus *Cetobacterium*.

At the genus level, the dominant bacterial genus in the domesticated and cultured groups was *Cetobacterium*, while the dominant bacterial genus in the wild group was *RsaHF231*, which may be due to differences in the water environment and food resources, leading to differences in the intestinal microbial species in the wild, domesticated, and cultured groups [44]. *Cetobacterium* synthesizes vitamin B12, essential for nucleic acid and protein biosynthesis [45,46]. It is also recognized as a beneficial probiotic associated with fish health [6,41], suggesting that the domesticated and cultured groups reared in recirculating aquaculture systems may exhibit enhanced disease resistance. These groups were fed artificial feed, known for its comprehensive nutritional composition, which likely promotes the colonization and proliferation of intestinal probiotics beneficial to fish health. Probiotics perform crucial functions in maintaining fish intestinal health by improving the composition of intestinal microbiota, suppressing the proliferation of pathogenic microorganisms, and modulating immune responses associated with the fish intestine [30,47].

Nevertheless, there was a certain amount of *Pseudomonas*, which is a pathogenic bacterium, in the cultured group, and *Pseudomonas* is more likely to infect a host with a disease [40]. There may be more organic matter in the culture environment, which easily causes water pollution and the proliferation of pathogenic bacteria. However, nutritional regulation and probiotic application may be a way to control the dynamic balance of the intestinal microbiota and prevent disease in the culture of *G. p. firmispinatus*.

### 4.3. Metabolic Function Analysis of the Intestinal Microbiota

The functional pathways of intestinal microbiota in *G. p. firmispinatus* primarily focus on biosynthetic activities. Specifically, pathways for amino acid synthesis, vitamin synthesis, and fatty acid and lipid synthesis were found to be more abundant. This result was similar to the results reported for *Coreius guichenoti* [43], *S. lalandi* [17], and *G. chilensis* [2]. These findings suggest that the intestinal microbiota of fish may play a vital role in amino acid and fatty acid synthesis [43].

Variations in the microbiota composition of *G. p. firmispinatus* between wild and farmed conditions could have substantial implications for the host, particularly concerning the functional pathways influenced by the microbiome components [17]. Specifically, the wild group showed higher abundance of the L-cysteine degradation III pathway (PWY-5329) compared to the domesticated group. Additionally, the vitamin E biosynthesis pathway (PWY-1422) was more abundant in the wild group than in the cultured group. The above results showed that the wild group exhibited greater amino acid degradation and vitamin synthesis.

Similarly, Romero et al. [2] noted that there were significant differences in the metabolic functional pathways of intestinal microbiota between wild and cultured *G. chilensis*. Specifically, under natural conditions, unsaturated fatty acid synthesis was important, whereas under aquaculture conditions, the metabolism of terpenoids and polyketides was relevant [2]. Similar results were observed for *P. adspersus* [16], *S. lalandi* [17], and *C. guichenoti* [43]. The different habitats and food resources could affect the bacterial composition, therefore influencing the function of the fish intestinal microbiota and providing feedback to the growth and health of fish [30].

## 5. Conclusions

The study revealed significant differences in the intestinal microbiome composition of wild, domesticated, and cultured *G. p. firmispinatus* at both the phylum and genus levels, illustrating the clear contrast between wild and farming conditions. The wild group exhibited higher richness and diversity in intestinal microbiota, with larger individual variations, reflecting greater ecological resilience. In contrast, the domesticated and cultured groups showed lower richness and diversity, with less variability among individuals. The intestinal microbial communities of these groups clustered distinctly based on their respective environments, with the domesticated and cultured groups showing greater similarity. Furthermore, our findings underscored the influence of different environments and food resources on the functional profiles of fish intestinal microbiota. The intestinal microbiome diversity and metabolic functionality in the wild group were structurally superior compared to those observed in the domesticated and cultured groups. The strategic use of probiotics has the potential to enhance the diversity of intestinal microbiota in *G. p. firmispinatus*, thereby maintaining a dynamic microbial balance crucial for disease prevention. Future research will emphasize regulating intestinal microbiota through the incorporation of probiotics into artificial compound feed and achieving a comprehensive understanding of the correlation between specific genera and their functional roles.

## Figures and Tables

**Table 1:** The composition of feed (wet weight, %, *n* = 3, ±SD).

Ingredient	Feed 2.0	Feed 4.0	Mealworm
Moisture	12.43 ± 0.01	12.45 ± 0.07	61.80 ± 1.96
Crude protein	42.00 ± 0.57	40.00 ± 0.77	18.94 ± 0.01
Crude fat	5.74 ± 0.04	5.31 ± 0.52	6.70 ± 0.10
Ash	11.74 ± 0.03	9.29 ± 0.05	5.59 ± 0.01

**Table 2:** Pairwise comparisons of *G. p. firmispinatus* intestinal microbiota based on Bray–Curtis distance and weighted UniFrac distance (*p*-Value). WG: wild group; CG: cultured group; DG: domesticated group.

Sample Source	Bray–Curtis		Weighted UniFrac	
	PERMANOVA	ANOSIM	PERMANOVA	ANOSIM
WG vs. DG	0.001	0.002	0.002	0.002
WG vs. CG	0.001	0.001	0.001	0.002
DG vs. CG	0.529	0.923	0.729	0.768

Note: non-significant: *p* > 0.05; significant: *p* < 0.05; highly significant: *p* < 0.01.

## Data Availability

The original contributions presented in this study are included in the article. Further inquiries can be directed to the corresponding authors.
